# Prolonged androgen deprivation leads to overexpression of calpain 2: Implications for prostate cancer progression

**DOI:** 10.3892/ijo.2013.2196

**Published:** 2013-11-29

**Authors:** TIANCHENG LIU, DESIREE E. MENDES, CLIFFORD E. BERKMAN

**Affiliations:** Department of Chemistry, Washington State University, Pullman, WA 99164, USA

**Keywords:** calpain 2, filamin A, androgen receptor, androgen deprivation, prostate cancer

## Abstract

Understanding the molecular mechanism of prostate cancer progression from androgen dependence to independence may lead to developing more effective treatments against prostate cancer. Herein, our previous *in vitro* model was employed to assess the effects of continuous androgen-deprivation on developing the metastatic phenotype from androgen-dependent prostate cancer cells (LNCaP). The results indicated that long-term androgen deprivation resulted in overexpression of calpain 2 and increased expression of filamin A (FlnA), but not for calpain 1. The enhanced activity of calpain 2 was confirmed by the accumulation of cleaved FlnA fragments, which could be effectively blocked by calpeptin (an inhibitor of calpain 2). Therefore, the combination of calpain 2 inhibitor and androgen deprivation may provide new therapeutic strategy for patients to prevent or postpone prostate cancer progression.

## Introduction

Prostate cancer remains the second leading cause of cancer death for men in the US. According to the National Cancer Institute, it is estimated that there will be 238,590 new cases and 29,720 deaths from prostate cancer in 2013 (http://www.cancer.gov/cancertopics/types/prostate). Initially, prostate cancer cells are dependent upon androgen stimulation for growth and proliferation, and thus sensitive to hormone therapy such as androgen deprivation, which effectively blocks androgen-dependent tumor growth. Unfortunately, most recurrent tumors return within two years exhibiting castration-resistant growth and a more aggressive, metastatic phenotype. As of yet, there is no effective treatment for castration-resistant prostate cancer (CRPC) (1,2). Therefore, it is of great urgency to elucidate and fully understand the molecular mechanisms of CRPC with a metastatic phenotype following androgen deprivation, in order to develop novel therapeutic strategies or approaches toward CRPC.

Recently, several mechanisms have been proposed for the development of castration-resistant prostate cancer (3,4) including mutation, amplification (4,5), expression of alternative-splice variants (6), proteolytic removal of ligand-binding domain (LBD) (7) of the androgen receptor (AR), or the increase of natural testosterone biosynthesis by cancer cells (8,9). These mechanisms suggest that most CRPC cells may still depend on AR function, but are adaptive to low hormone levels or enable AR function for independence of ligand-binding. However, clinical evidence and basic research study support the hypothesis that alternative mechanisms may be related to highly aggressive, metastatic phenotype in AR-negative prostate cancer cells or cancer-stem cells (10–14).

Calpains are a family of calcium-dependent, non-lysosomal cysteine proteases, of which 14 human calpains are well known. Based on structure and function, calpain 1 (μ-calpain) and calpain 2 (m-calpain) are well characterized (15). There is a plenty of experimental and clinical evidence to support critical roles of calpains in normal cellular functions (cell motility, cell cycle, autophagy and apoptosis), and pathological processes (tumorigenesis, cancer metastasis and neurodegenerative diseases) (15,16). In fact, overexpression of calpain 2 has been confirmed in human colorectal cancer (17), and increased expression of calpain 1 in schwannomas and meningiomas has also been reported (18). However, results for prostate cancer are controversial (19,20). Filamin A (FlnA), also known as actin-binding protein 280 (ABP-280), is a 280 kDa protein consisting of N-terminal actin-binding domain (ABD) and a rodlike domain of 24 repeats (each repeat is approximately 96 amino acids in length), which is interrupted by two hinge domains denoted as H1 (between repeats 15 and 16) and H2 (between repeats 23 and 24). FlnA is a natural substrate of calpains 1/2 in cells and cleavage occurs at H1 resulting in two fragments of 170 and 110 kDa, respectively. The 110 kDa fragment containing the H2 domain, can be further cleaved to yield a 90 kDa fragment which has been confirmed to translocate to the nucleus and bind AR in androgen-sensitive LNCaP cells, with nuclear translocation absent in AR-negative PC-3 cells (21). The dimerization domain (65 amino acids located in repeat 24) of FlnA forms a structure-shaped homodimer motif to bind or cross-link actin filaments of cytoskeleton networks through its N-terminal ABD domain. In addition, FlnA can serve as a molecular scaffold interacting with multiple proteins such as transmembrane proteins, signaling molecules and DNA damage repair proteins (22). Recent studies demonstrated that an increase in both expression and cytoplasmic distribution of FlnA was related to aggressive, metastatic prostate cancer (23). A new discovery revealed that calpain 2 can cleave and remove the ligand-binding domain (LBD) from AR. In turn, the truncated AR retaining the transactivation domain and DNA-binding domain (DBD), exhibited a constitutively active AR molecule that translocated into the nucleus and functioned in an androgen-independent manner (7,24).

Our previous studies revealed the occurrence of two important events in prostate cancer cells which were the loss of prostate specific membrane antigen (PSMA) expression and a cell signal pathway switch from AR to c-Met during long-term androgen deprivation in an established *in vitro* model (10,25). Herein we further explore whether long-term androgen deprivation can upregulate the expression and activity of calpains 1/2 in prostate cancer cells, which are correlated with a highly aggressive, metastatic phenotype (20). Our present data strongly support the concept that long-term androgen deprivation may push androgen-sensitive prostate cancer cells evolve into AR-negative, more aggressive, androgen-independent disease state, with overexpression of calpain 2 enhancing its activity. Thus, a combination of calpain 1/2 inhibitor and androgen deprivation may provide a novel therapeutic strategy to prevent or postpone the progression of prostate cancer from androgen-sensitive to CRPC.

## Materials and methods

### Cell lines and reagents

The human prostate cancer cell lines LNCaP and PC-3 were obtained from the American Type Culture Collection (Manassas, VA, USA). Rabbit polyclonal anti-AR antibody (N-20) was obtained from Santa Cruz Biotechnology (Santa Cruz, CA, USA). Mouse monoclonal anti-FlnA (N-terminus) antibody, goat anti-rabbit secondary antibody-FITC, and rabbit anti-actin antibody were obtained from Sigma-Aldrich (St. Louis, MO, USA). Protein blocking solution was obtained from BioGenex (San Ramon, CA, USA). Rabbit polyclonal anti-calpain 1 and anti-calpain 2 antibodies were obtained from Cell Signaling Technology (Danvers, MA, USA). Mouse monoclonal anti-FlnA (C-terminus) antibody and calpeptin were obtained from EMD Millipore Corporation (Billerica, MA, USA). Hoechst 33342 were obtained from Invitrogen-Molecular Probes (Carlsbad, CA, USA). Halt Protease Inhibitor Cocktail 100X was purchased from Thermo Fisher Scientific (Rockford, IL, USA). All other chemicals and cell-culture reagents were purchased from Fisher Scientific (Sommerville, NJ, USA) or Sigma-Aldrich.

### Cell culture

LNCaP and PC-3 cells were grown in T-75 flasks with normal growth media [RPMI-1640 containing 10% heat-inactivated fetal calf serum (FBS), 100 units of penicillin and 100 *μ*g/ml streptomycin] in a humidified incubator at 37°C with 5% CO_2_. Otherwise, for androgen-deprivation growth, cells were cultured with conditioned media [RPMI-1640 containing 10% charcoal-stripped fetal bovine serum, 100 units of penicillin and 100 *μ*g/ml streptomycin]. Confluent cells were detached with a 0.25% trypsin 0.53 mM EDTA solution, harvested, and plated in 2-well slide chambers at a density of 4×10^4^ cells/well. Cells were grown for three days before conducting the following experiments.

### Immunofluorescence detection of AR and FlnA

The LNCaP cells grown under androgen deprivation condition over time (5, 10 and 20 passages) were cultured for 3 days on the slides in the conditioned media. For two-day androgen-deprivation treatment, LNCaP cells were seeded on slides with normal growth media for 1-day growth, and replaced with conditioned media for another 2-day growth. Normal LNCaP cells and PC-3 cells were used for the AR-positive and AR-negative control, respectively. These cells were seeded on slides with normal growth media for three days. Slides with cells grown for three days in normal growth media or conditioned media were washed twice in phosphate-buffered saline (PBS), fixed in 4% paraformaldehyde in PBS buffer for 15 min at room temperature, and permeabilized with pre-cooled methanol for 5 min at −20°C. The permeabilized cells were blocked in block buffer (0.1% Tween-20, 5% goat normal serum in PBS buffer) for 2 h at room temperature and incubated with primary anti-AR antibody and anti-FlnA (C-terminus) antibody in block buffer overnight at 4°C. After washing, the cells were incubated with a secondary antibodies (goat anti-rabbit IgG-FITC and goat anti-mouse IgG-TRITC in 1% BSA, PBS buffer) for 2 h at room temperature, counterstained with Hoechst 33342, and mounted in VECTASHIELD^®^ Mounting Medium (Vector Laboratories, Burlingame, CA, USA) for confocal microscopy.

### Confocal laser scanning microscopy

Cells were visualized under 25X water immersion objective using an LSM 510 META Laser Scanning Microscope. Hoechst 33342 was excited with a Diode Laser (405 nm), and the emission was collected with a BP420-480 nm filter. AR immunofluorescence (with goat anti-rabbit IgG-FITC) was excited at 488 nm using an Argon Laser, and the emission was collected with an LP505 nm filter. FlnA immunofluorescence (with goat anti-mouse IgG-TRITC) was excited using 543 nm from a HeNe Laser, and the emission collected with a BP560-615 nm filter. To reduce interchannel crosstalk, a multi-tracking technique was used, and images were taken at a resolution of 1,024×1,024 pixels. Confocal scanning parameters were set up so that the control cells without treatment did not display background fluorescence. The imaging colors of the fluorescent dyes, Hoechst 33342, FITC and TRITC, were defined as blue, green and red, respectively. The pictures were edited by National Institutes of Health (NIH) Image J software (http://rsb.info.nih.gov/ij) and Adobe Photoshop CS2.

### Whole cell lysate extraction and western blot analysis

The controls: PC-3 and LNCaP cells (cultured in normal growth media) and LNCaP cells under androgen deprivation over time (2 days, 5, 10 or 20 passages) were collected by scraping, washed once in ice-cold PBS, re-suspended in 3-fold cell pellet volumes of lysis buffer (1% NP-40, 20 mM Tris pH 8.0, 137 mM NaCl, 10% glycerol) (10,25–27) supplemented with 1X Halt Protease Inhibitor Cocktail for 15 min on ice, then transferred to Eppendorf tubes for centrifugation at 10,000 × g for 15 min at 4°C, the supernatant was saved as whole-cell protein extracts. For calpain 1/2 inhibitor block study, LNCaP cells under androgen deprivation over time (5, 10 passages) and PC-3 cells were allowed to be cultured for 2 days, then continued for another 24 h growth in the above mentioned media containing 40 *μ*M calpeptin or DMSO, prior to cell harvest and protein extraction. Protein concentrations were determined using Non-Interfering Protein Assay (G-Biosciences, St. Louis, MO, USA). Western blot analysis was performed as described previously with only minor modifications (27,28). In brief, detergent-soluble proteins (30 *μ*g) were loaded and separated on a NuPAGE™ 4-12% Bis-Tris Gel (Invitrogen, Carlsbad, CA, USA) by electrophoresis for 60 min at a constant 200 V under reducing conditions, and then transferred to a 0.45 *μ*m PVDF Immobilon-P Transfer Membrane (Millipore Corporation) at 400 mA for 120 min in a transfer apparatus-Owl Bandit VEP-2 (Owl, Portsmouth, NH, USA) according to the manufacturer’s instructions. Membranes were incubated with primary antibody at corresponding dilution overnight at 4°C and then with horse-radish peroxidase conjugated-second antibody for 1 h at room temperature. The immunoreactive bands were visualized using Protein Detector TMB Western Blot kit (KPL, Gaithersburg, MD, USA) following the manufacturer’s instructions.

## Results

### Overexpression of calpain 2, not calpain 1 induced by androgen deprivation

**Figure 1.**
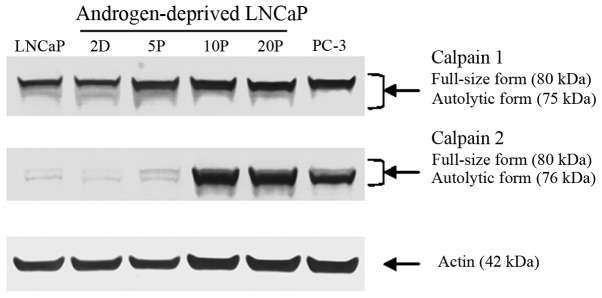
Western blot detection of calpain 1 and calpain 2 expression. There is overexpression of calpain 2 in long-term androgen-deprived LNCaP cells (10, 20 passages) and PC-3 cells, compared to LNCaP cells cultured in normal growth medium, and short-term androgen deprivation (2 days, 5 passages). There is no significant change for calpain 1 expression in all LNCaP cells proliferated under normal or androgen deprivation conditions and PC-3 cells. Actin expression was detected to serve as protein loading controls.

Western blot analysis (Fig. 1) clearly demonstrated that overexpression of calpain 2 was observed in long-term androgen-deprived LNCaP cells (10, 20 passages) and PC-3 cells (AR-negative), of which, AR was confirmed to be no longer detectable in our previous work (10,25). In contrast, calpain 1 expression was relatively stable in both AR-positive and AR-negative prostate cancer cells.

### Increased expression and proteolytic cleavage of FlnA

**Figure 2.**
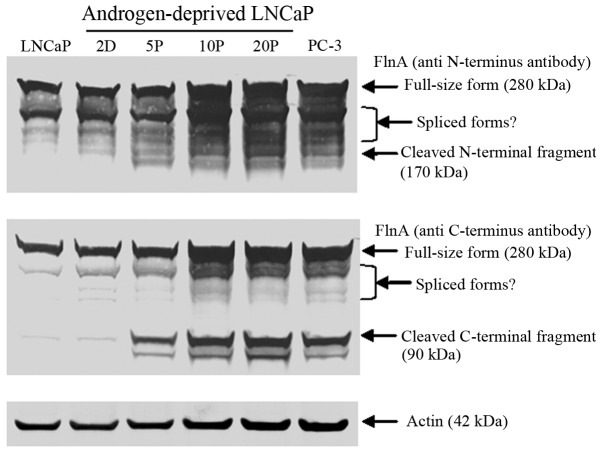
Western blot detection of FlnA expression and its cleaved fragments. By employment of anti-FlnA C-terminus or N-terminus antibodies, increased expression of FlnA and accumulation of cleaved fragments were detected in LNCaP cells proliferated under androgen deprivation conditions by 10 passages (10P), 20 passages (20P) and PC-3 cells. Actin expression was detected to serve as a protein loading controls.

Western blot analysis also revealed that there was an apparent increase of FlnA expression and accumulation of its cleaved fragments (Fig. 2) in long-term androgen-deprived LNCaP cells (10, 20 passages) and AR-negative PC-3 cells (10,25). The increase of cleaved FlnA fragments (Fig. 2) is clearly correlated with overexpressed calpain 2 in these cells (Fig. 1).

### Calpeptin protected FlnA from cleavage in AR-negative cells

**Figure 3.**
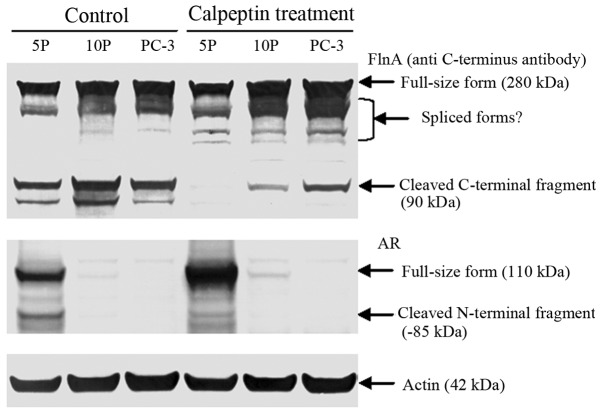
Protection of FlnA and AR from calpain-mediated cleavage in calpeptin-treated cells. By employment of anti-FlnA C-terminus antibodies, there was apparent decrease of cleaved FlnA 90 kDa fragment in LNCaP cells proliferated under androgen deprivation conditions by 5 passages (5P), 10 passages (10P) and AR-negative PC-3 cells, compared to control cells. In the same manner, AR was also clearly protected from proteolysis with increase of full-size and decrease of truncated 85 kDa fragment in LNCaP cells proliferated under androgen deprivation conditions by 5 passages (5P). It is clear that inhibition of calpain 1/2 by calpeptin strongly protected FlnA and AR from calpain-mediated cleavage. Actin expression was detected to serve as protein loading controls.

Calpain-block study in cells further confirmed that calpeptin treatment effectively inhibited calpain 1/2 activities, and protected FlnA from cleavage (Fig. 3). Through cleavage of FlnA, calpain 2 can dynamically regulate FlnA mediated cytoskeleton network, and further regulate cell signal pathways, cell morphology and motility.

### Calpeptin protected AR from cleavage

In Fig. 3, compared to control cells, it was also noticed that AR was clearly protected from calpain-mediated cleavage by calpeptin treatment in relatively short-term androgen-deprived LNCaP cells (5 passages). An increase of 85 kDa truncated AR fragment with removal of ligand-binding domain in CWR22Rv1 cells, has been suggested to be a mechanism for androgen independence of prostate cancer cells (7).

### Different distribution of FlnA in AR-positive and AR-negative cells

**Figure 4.**
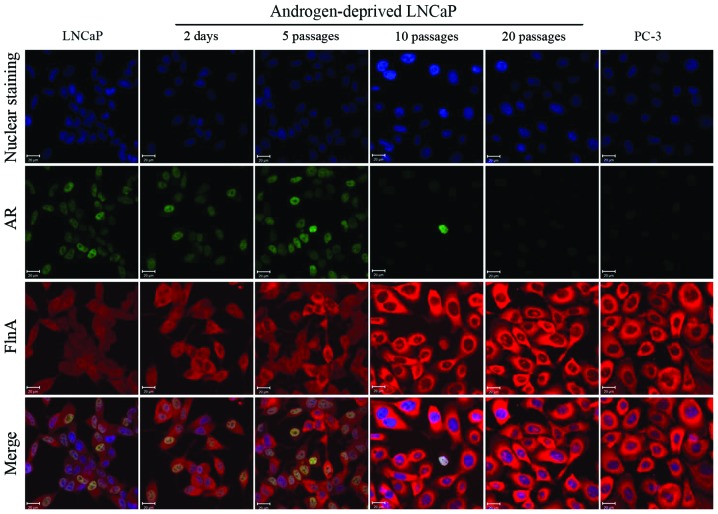
Immunofluorescence detection of both AR and FlnA expression in LNCaP cells following androgen deprivation treatment. Specific fluorescence signals for AR (green) were detected throughout the nuclei in normal LNCaP cells and LNCaP cells under androgen deprivation conditions for 2 days and 5 passages. No signals for AR were observed for LNCaP cells proliferated under androgen deprivation conditions by 10 passages or the AR-negative PC-3 cells. In contrast, fluorescence signals for expression-increased FlnA (red) were detected in LNCaP cells proliferated under androgen deprivation conditions by 10 passages or PC-3 cells. Cell nuclei were counterstained with Hoechst 33342 (blue). Cellular imaging was visualized by confocal microscopy; distance scale is 20 *μ*m.

A relatively average distribution of FlnA from the cytoplasm to the nucleus, strong nuclear co-localization of AR and FlnA, and a slight increase of cytoplasmic distribution of FlnA over time were observed in AR-positive cells (Fig. 4). Inversely, the cytoplasmic distribution of FlnA was the highest in AR-negative cells (Fig. 4). It was also noticable that a minimal amount of FlnA was located within the nucleolus in AR-negative cells (Fig. 4). The contrasting distribution of FlnA in the nucleus or in the cytoplasm is consistent with clinical data in which immunohistochemistry analysis demonstrated that cytoplasmic localization of FlnA is highly correlated with metastases of prostate cancer (23).

## Discussion

Our previous study suggests that long-term androgen depletion may induce a signaling pathway switch from AR to c-Met, which may lead to a diagnostically and therapeutically elusive androgen-independent disease-state. Following our previous research studies, our present data further define the key role of calpain 2 and not calpain 1 in prostate cancer progression, especially in cancer invasion and metastasis with its overexpression and enhanced activity in AR-negative prostate cancer cells.

Both calpains 1 and 2 are heterodimers, which consist of a specific 80 kDa catalytic subunit and a common regulatory 28 kDa subunit shared by calpain 1 and calpain 2. Their proteolytic activities exhibit dependence on different calcium concentrations *in vitro* (15). Therefore, activation of calpain 1 or calpain 2 may be responsible for different signaling pathways and physiological or pathological processes in cells. Calpastatin is an endogenous inhibitor of calpain 1 and calpain 2 while inhibitory action of calpastatin is also dependent on calcium-induced structural changes of calpain 1/2 (15). Calcium, calpastatin and calpain 1/2 are three components whose concentration, distribution and interaction determine spatial and temporal regulation of calpain 1/2 activity in cells (15). The dynamic regulation of calpain activity is necessary for coordination of cell-substrate adhesion, actin and myosin-mediated contraction and cell-substrate detachment to control cell movement (15). There is experimental evidence that demonstrates calpain 1/2 has several roles in cancer progression such as cleaving focal adhesion kinase (FAK) to dynamically regulate integrin-mediated focal adhesion for cell migration (29,30), regulating activation of membrane-type matrix metalloproteinase 1 (MT1-MMP or MMP-14) and matrix metalloproteinase 2 (MMP2) for extra-cellular matrix remodeling and angiogenesis (31), and cancer invasion and metastasis (32,33).

Considering the multiple cellular functions of calpain 2, its abnormal high expression and enhanced activity may play important roles in prostate cancer progression including migration, invasion and metastasis during androgen deprivation therapy. It is reasonable that calpain 2 may be treated as a target for limiting tumor progression. In fact, inhibition or downregulation of calpain 2 clearly decreased migration and invasion of DU-145 prostate cancer cells *in vitro* and *in vivo* (19). Furthermore, calpain 2 can also cleave AR to generate a truncated, functional AR without ligand-binding domain in androgen-sensitive prostate cancer cells, which enables cancer cell adaptation to androgen-independent growth and proliferation during androgen-deprivation treatment (7). Our present study further confirms the above discovery in short-term androgen-deprived LNCaP cells (5 passages). It should be mentioned that short-term androgen-deprived LNCaP cells (5 passages) can survive without androgen in media, but grow and proliferate at very low rates (10). In contrast, long-term androgen deprivation caused the most loss of AR expression, with development of alternative signaling pathways enabling cell growth and proliferation at high rates (10,25). In addition, inhibition of calpain activity can enhance cytotoxic activity of bortezomib *in vitro* and *in vivo* against cancer cells by preventing autophagic survival response (34).

In summary, our present data support the concept that long-term androgen deprivation promotes overexpression and enhanced activity of calpain 2 leading to an increase in the fragmental cleavage of AR and FlnA. The overexpression of calpain 2 and increased expression of FlnA may contribute to the development of an aggressive phenotype of prostate cancer. It is expected that the combination of androgen deprivation with inhibition of calpain 2 may provide a new therapeutic strategy to prevent or postpone appearance of CRPC in patients.

## References

[1] Gustavsson H, Welen K, Damber JE (2005). Transition of an androgen-dependent human prostate cancer cell line into an androgen-independent subline is associated with increased angiogenesis. Prostate.

[2] Niu Y, Altuwaijri S, Lai KP (2008). Androgen receptor is a tumor suppressor and proliferator in prostate cancer. Proc Natl Acad Sci USA.

[3] Lee SO, Dutt SS, Nadiminty N, Pinder E, Liao H, Gao AC (2007). Development of an androgen-deprivation induced and androgen suppressed human prostate cancer cell line. Prostate.

[4] Saraon P, Jarvi K, Diamandis EP (2011). Molecular alterations during progression of prostate cancer to androgen independence. Clin Chem.

[5] Devlin HL, Mudryj M (2009). Progression of prostate cancer: multiple pathways to androgen independence. Cancer Lett.

[6] Watson PA, Chen YF, Balbas MD (2010). Constitutively active androgen receptor splice variants expressed in castration-resistant prostate cancer require full-length androgen receptor. Proc Natl Acad Sci USA.

[7] Libertini SJ, Tepper CG, Rodriguez V, Asmuth DM, Kung HJ, Mudryj M (2007). Evidence for calpain-mediated androgen receptor cleavage as a mechanism for androgen independence. Cancer Res.

[8] Cai C, Balk SP (2011). Intratumoral androgen biosynthesis in prostate cancer pathogenesis and response to therapy. Endocr Relat Cancer.

[9] Chang KH, Li R, Kuri B (2013). A gain-of-function mutation in DHT synthesis in castration-resistant prostate cancer. Cell.

[10] Liu T, Mendes DE, Berkman CE (2013). From AR to c-Met: Androgen deprivation leads to a signaling pathway switch in prostate cancer cells. Int J Oncol.

[11] Hendriksen PJ, Dits NF, Kokame K (2006). Evolution of the androgen receptor pathway during progression of prostate cancer. Cancer Res.

[12] Lorenzo GD, Bianco R, Tortora G, Ciardiello F (2003). Involvement of growth factor receptors of the epidermal growth factor receptor family in prostate cancer development and progression to androgen independence. Clin Prostate Cancer.

[13] Maeda A, Nakashiro K, Hara S (2006). Inactivation of AR activates HGF/c-Met system in human prostatic carcinoma cells. Biochem Biophys Res Commun.

[14] Nishida S, Hirohashi Y, Torigoe T (2013). Prostate cancer stem-like cells/cancer-initiating cells have an autocrine system of hepatocyte growth factor. Cancer Sci.

[15] Storr SJ, Carragher NO, Frame MC, Parr T, Martin SG (2011). The calpain system and cancer. Nat Rev Cancer.

[16] Camins A, Verdaguer E, Folch J, Pallas M (2006). Involvement of calpain activation in neurodegenerative processes. CNS Drug Rev.

[17] Lakshmikuttyamma A, Selvakumar P, Kanthan R, Kanthan SC, Sharma RK (2004). Overexpression of m-calpain in human colorectal adenocarcinomas. Cancer Epidemiol Biomarkers Prev.

[18] Kimura Y, Koga H, Araki N (1998). The involvement of calpain-dependent proteolysis of the tumor suppressor NF2 (merlin) in schwannomas and meningiomas. Nat Med.

[19] Mamoune A, Luo JH, Lauffenburger DA, Wells A (2003). Calpain-2 as a target for limiting prostate cancer invasion. Cancer Res.

[20] Rios-Doria J, Day KC, Kuefer R (2003). The role of calpain in the proteolytic cleavage of E-cadherin in prostate and mammary epithelial cells. J Biol Chem.

[21] Wang Y, Kreisberg JI, Bedolla RG, Mikhailova M, deVere White RW, Ghosh PM (2007). A 90 kDa fragment of filamin A promotes Casodex-induced growth inhibition in Casodex-resistant androgen receptor positive C4-2 prostate cancer cells. Oncogene.

[22] Yue J, Huhn S, Shen Z (2013). Complex roles of filamin-A mediated cytoskeleton network in cancer progression. Cell Biosci.

[23] Bedolla RG, Wang Y, Asuncion A (2009). Nuclear versus cytoplasmic localization of filamin A in prostate cancer: immunohistochemical correlation with metastases. Clin Cancer Res.

[24] Chen H, Libertini SJ, Wang Y, Kung HJ, Ghosh P, Mudryj M (2010). ERK regulates calpain 2-induced androgen receptor proteolysis in CWR22 relapsed prostate tumor cell lines. J Biol Chem.

[25] Liu T, Wu LY, Fulton MD, Johnson JM, Berkman CE (2012). Prolonged androgen deprivation leads to downregulation of androgen receptor and prostate-specific membrane antigen in prostate cancer cells. Int J Oncol.

[26] Matroule JY, Carthy CM, Granville DJ, Jolois O, Hunt DW, Piette J (2001). Mechanism of colon cancer cell apoptosis mediated by pyropheophorbide-a methylester photosensitization. Oncogene.

[27] Liu T, Wu LY, Berkman CE (2010). Prostate-specific membrane antigen-targeted photodynamic therapy induces rapid cytoskeletal disruption. Cancer Lett.

[28] Liu T, Toriyabe Y, Berkman CE (2006). Purification of prostate-specific membrane antigen using conformational epitope-specific antibody-affinity chromatography. Protein Expr Purif.

[29] Chan KT, Bennin DA, Huttenlocher A (2010). Regulation of adhesion dynamics by calpain-mediated proteolysis of focal adhesion kinase (FAK). J Biol Chem.

[30] Franco SJ, Rodgers MA, Perrin BJ (2004). Calpain-mediated proteolysis of talin regulates adhesion dynamics. Nat Cell Biol.

[31] Kwak HI, Kang H, Dave JM (2012). Calpain-mediated vimentin cleavage occurs upstream of MT1-MMP membrane translocation to facilitate endothelial sprout initiation. Angiogenesis.

[32] Jang HS, Lal S, Greenwood JA (2010). Calpain 2 is required for glioblastoma cell invasion: regulation of matrix metalloproteinase 2. Neurochem Res.

[33] Postovit LM, Dutt P, Dourdin N (2002). Calpain is required for MMP-2 and u-PA expression in SV40 large T-antigen-immortalized cells. Biochem Biophys Res Commun.

[34] Escalante AM, McGrath RT, Karolak MR, Dorr RT, Lynch RM, Landowski TH (2013). Preventing the autophagic survival response by inhibition of calpain enhances the cytotoxic activity of bortezomib in vitro and in vivo. Cancer Chemother Pharmacol.

